# Forty-Ninth Annual Session of the Medical Association of Georgia

**Published:** 1898-04

**Authors:** 


### Society Reports.

---

### FORTY-NINTH ANNUAL SESSION OF THE MEDICAL ASSOCIATION OF GEORGIA.

WEDNESDAY, April 28, 1898.

1. Treatment of Typhoid Fever.—Wm. J. Cox, M. D., Barnesville.
2. A Report of Surgical Cases.—Hunter P. Cooper, M. D., Atlanta.
3. Malarial Asthenopia.—J. Lawton Heirs, M. D., Savannah.
4. Diphtheria Antitoxin with Report of Cases.—Henry R. Slack, M. D., LaGrange.
5. Solid Tumors of the Ovary.—Virgil O. Hardon, M. D., Atlanta.
6. Puerperal Eclampsia and Some Probable Causes of it.—J. I. Griffeth, M. D., Danielsville.
7. Hysteria.—A. A. Davidson, M. D., Augusta.
8. Femoral Hernia with Report of Cases.—Floyd W. McRae, M. D., Atlanta.
9. Primary Sarcoma of the Orbit, with Cases.—Alex. W. Stirling, M. D., Atlanta.
10. Mushrooms, a Food and a Poison.—W. H. Elliott, M. D., Savannah.
11. The Indication for, and Antiseptic Technique of Uterine Drainage, after Labor and Abortion.—R. R. Kime, M. D., Atlanta.
12. A Rare Form of Bone Atrophy following an Ununited Fracture, as seen by the X-ray.—Eugene R. Corson, M. D., Savannah.
13. Surgical Treatment of Hemorrhoids.—Wm. S. Goldsmith, M. D., Atlanta.
14. A Few Cases from my Note-Book.—J. G. Hopkins, M. D., Thomasville.
15. The Possibilities of a Perfect Aseptic Surgical Technique.—E. C. Davis, M. D., Atlanta.
16. Diphtheria; its Present Status and Treatment.—B. C. Powell, M. D., Villa Rica.
17. Locomotor Ataxia.—Hugh Hagan, M. D., Atlanta.
18. Shall Georgia Have a Medical Law Pertaining to Marriage Contracts?—J. Cheston King, M. D., Newnan.
19. Tonsilotomy, When and How to Make it.—J. M. Crawford, M. D., Atlanta.
20. A Georgia Incubator.—W. P. Williams, M. D., Blackshear.

21. Some Remarks on { (a) Appendicitis, (b) Hernia. }—W. F. Westmoreland, M. D., Atlanta.

22. Chronic Diarrhœa as an Entity, with Report of Cases.—T. M. Greenwood, M. D., Mineral Bluff.

23. A Few Remarks on the Treatment of Post Nasal Adenitis.—M. F. Carson, M. D., Griffin.

24. The Physician.—J. S. Wimberly, M. D., Sunlight.

25. The Endoscopic Treatment of Chronic Urethritis, with Report of Cases.—W. L. Champion, M. D., Atlanta.

26. Antitoxin in the Treatment of Diphtheria.—B. L. Bridges, M. D., Ellaville.

27. Adenoids in the Pharyngeal Vault, and Their Influence Upon the Ear.—A. W. Calhoun, M. D., Atlanta.

28. The Deadbeat vs. the Doctor.—W. J. Rowe, M. D., Buford.

29. Report of a Case of Poliomyelitis.—W. L. Patten, M. D., Milltown.

30. Some Pathological Investigations.—Chas. M. Blackford, M. D., Augusta.

31. (Title to be furnished.)—S. Latimer Phillips, M. D., Savannah.

32. Alcoholic Irrigation in Puerperal Sepsis.—George H. Noble, M. D., Atlanta.

33. The Importance of Careful Chemical Analysis in Gastric Disorders.—Wm. Clifton Lyle, M. D., Augusta.

